# Effect of staining solutions and repolishing on color stability of direct composites

**DOI:** 10.1590/S1678-77572010000300009

**Published:** 2010

**Authors:** Fabrício Mariano MUNDIM, Lucas da Fonseca Roberti GARCIA, Fernanda de Carvalho Panzeri PIRES-DE-SOUZA

**Affiliations:** 1 DDS, MSc, PhD student, Department of Dental Materials and Prosthodontics, Ribeirão Preto Dental School, University of São Paulo, Ribeirão Preto, SP, Brazil.; 2 DDS, MSc, PhD, Researcher, Department of Dental Materials and Prosthodontics, Ribeirão Preto Dental School, University of São Paulo, Ribeirão Preto, SP, Brazil.; 3 DDS, MSc, PhD, Associate Professor, Department of Dental Materials and Prosthodontics, Ribeirão Preto Dental School, University of São Paulo, Ribeirão Preto, SP, Brazil.

**Keywords:** Composite resins, Color, Spectrophotometry, Dental polishing

## Abstract

**Objectives:**

The purpose of this study was to assess the color change of three types of composite resins exposed to coffee and cola drink, and the effect of repolishing on the color stability of these composites after staining.

**Materials and Methods:**

Fifteen specimens (15 mm diameter and 2 mm thick) were fabricated from microhybrid (Esthet-X; Dentsply and Filtek Z-250; 3M ESPE) and high-density hybrid (Surefil; Dentsply) composites, and were finished and polished with aluminum oxide discs (Sof-Lex; 3M ESPE). Color of the specimens was measured according to the CIE L*a*b* system in a reflection spectrophotometer (PCB 6807; BYK Gardner). After baseline color measurements, 5 specimens of each resin were immersed in different staining solutions for 15 days: G1 - distilled water (control), G2 - coffee, G3 - cola soft drink. Afterwards, new color measurement was performed and the specimens were repolished and submitted to new color reading. Color stability was determined by the difference (∆) between the coordinates L*, a*, and b* obtained from the specimens before and after immersion into the solutions and after repolishing.

**Results:**

There was no statistically significant difference (ANOVA, Tukey's test; p>0.05) among the ∆ values for the different types of composites after staining or repolishing. For all composite resins, coffee promoted more color change (∆>3.3) than distilled water and the cola soft drink. After repolishing, the ∆ values of the specimens immersed in coffee decreased to clinically acceptable values (∆<3.3), but remained significantly higher than those of the other groups.

**Conclusions:**

No significant difference was found among composite resins or between color values before and after repolishing of specimens immersed in distilled water and cola. Immersing specimens in coffee caused greater color change in all types of composite resins tested in this study and repolishing contributed to decrease staining to clinically acceptable ∆ values.

## INTRODUCTION

The problem of composite resin color change is well acknowledged by dentists^5^. An esthetic restoration with an unacceptable color is the main cause for replacement of anterior tooth restorations^19^. Color change usually occurs due to three reasons: 1) external discolorations due to accumulation of plaque and stains; 2) alterations on the surface or subsurface, promoting surface degradation and slight penetration and reaction of staining agents with the composite surface (adsorption); 3) intrinsic discolorations due to physicochemical reactions in the deep portions of the restoration^2^.

Composite structure and the characteristics of the inorganic fillers have a direct impact on resin surface smoothness^25^ and susceptibility to extrinsic staining^21^. In addition to the material’s composition, finishing and polishing procedures may influence the quality of the composite surface and can therefore be related to the early discoloration of resins^21^. The probability of stain penetration into its resin matrix is lower in smoother and polished composite surface than in rough surfaces. Several studies have been conducted to determine the effects of staining solutions on the surface characteristics of esthetic restorative materials^19,30^ , although the effectiveness of repolishing on the stains removal has not been widely investigated.

Dentists are routinely questioned by patients about how long an esthetic restoration should last, and if their eating habits may influence the quality and longevity of the restoration. The consumption of coffee and soft drinks, for example, has a high prevalence in the contemporary society, especially in industrialized countries. It has been demonstrated that surface discolorations in composite resins are related to hygiene, eating habits and smoking^2^. The maintenance of the esthetics of a restoration is therefore related to the patients’ habits and lifestyle.

Several types of composites are available for esthetic restorations of anterior and posterior teeth, and differ from each other according to the type of resin matrix, size, type, and amount of filler particles. Together, these characteristics may affect the esthetic properties of these materials. Repolishing may be a viable option to recover the esthetics of non-severely stained composite restorations^12^. Thus, the purpose of this study was to assess color change of three types of composite resins exposed to coffee and cola drink as well as the effect of repolishing on the color stability of these composite resins after staining. The tested null hypotheses were that there is no difference in the color stability of composites after immersion in staining solutions and repolishing.

## MATERIAL AND METHODS

Three direct composite resins currently indicated for esthetic anterior and/or posterior restorations were used in the present study. Information regarding composite type, composition, curing time and manufacturer is given in Table 1.

**Table 1:** Tested materials

**Commercial name**	**Type**		**Composition**		**Curing time**	**Manufacturer**
**Fillers**						
		**Monomers**	**Size (μm)**	**Volume %**		
						
Esthet-X	Microhybrid composite	Bis-GMA Bis-EMA TEGDMA	0.04-1	60%	20 s	Dentsply/Caulk, Milford, DE, USA
						
SureFil	High-density hybrid (packable) composite	Bis-GMA UDMA	0.8	65%	40 s	Dentsply DeTrey GmbH, Konstanz, Germany
						
Filtek-Z250	Microhybrid composite	Bis-GMA UDMA Bis-EMA	0.01-3.5	60%	20 s	3M ESPE, St. Paul, MN, USA

Bis-GMA, Bisphenol A diglycidyl ether dimethacrylate; Bis-EMA, Ethoxylated bisphenol A dimethacrylate, TEGDMA, Triethylene glycol dimethacrylate; UDMA, urethane dimethacrylate

Fifteen specimens (15 mm diameter x 2 mm thick) of each composite were fabricated using a stainless steel matrix. each material was inserted into the matrix in 1.0-mm-thick increments photoactivated with a halogen light-curing unit (Ultralux, Dabi Atlante, Ribeirão Preto, SP, Brazil), according to the manufacturer’s information (Table 1). The specimens were polished with aluminum oxide discs (Sof-Lex, 3M eSPe, St. Paul, MN, USA) in a sequence of decreasing abrasiveness with intermittent movements, and the specimen surface was kept moist at each disc change. The polished specimens had their thickness measured with an electronic digital caliper accurate to 0.1 mm (Digimess, São Paulo, SP, Brazil). The polished specimens were stored in the dark at 100% of humidity for 24 h.

Color was measured according to the CIe (Commission Internationale de l´eclairage) L*a*b* system relative to CIe standard illuminant D65, against a white background (Standard for 45/0 degrees; Gardner Laboratory, Inc, Bethesda, MD, USA) in a reflection spectrophotometer (PCB 6807 BYK Gardner, Geretsried, Germany). This equipment is specific for color measurement and has 30 LED lamps with 10 different colors arranged in a circle, which directs a light bundle at 45º with the material surface. This light bundle is reflected 0º back to the equipment, which captures and records the L*, a* and b* values of each specimen. The axis L* refers to the lightness coordinate and its value ranges from zero (black) to 100 (white). The axes a* and b* are chromaticity coordinates in the red-green axis and the yellow-blue axis, respectively. Positive a* values indicate a shift to red and negative values indicate a shift to green. Similarly, positive b* values indicate the yellow color range and negative values indicate the blue color range.

After baseline color measurement, the specimens were assigned to three groups (n=5), each one immersed in a different solution, and subjected to a new color measurement. Group 1 (control) was immersed in distilled water; Group 2 was immersed in coffee prepared by dissolving 1.5 g of soluble coffee (Nescafé Classic; Nestlé SA, Vevey, Switzerland) in 50 mL of boiling distilled water, and Group 3 was immersed in a cola soft drink (Coca-Cola^®^, Refrescos Ipiranga, Ribeirão Preto, SP, Brazil). The solutions were replaced every day.

After 15 days immersed in the solutions, the specimens were rinsed with distilled water for 5 min and blotted dry with absorbent paper before the second color measurement. Color of the specimens was measured after immersion in the different solutions by the spectrophotometer, as previously described. Using the values of L*, a*, b*, color variation (De1) between 15-day immersion and baseline measurements was determined using the following equation:

ΔE1 = [ (ΔL*) ^2^ + (Δa*) ^2^ + (Δb*)^2^]½

The values of ∆ > 1 are considered as visually perceptible and values of DE ≥ 3.3 are considered as clinically unacceptable^18^. After determination of color variation (De1), the specimens were repolished with Sof-Lex discs (3M ESPE) as previously described in the first polishing procedure. The repolished specimens were submitted to a new color measurement (CIe L*a*b*) and these values were compared to those of the baseline color measurements, so a second value of color variation (∆2) was obtained.

The means and standard deviations of color change (∆1 and ∆2) were calculated and submitted to statistical analysis by 3-way repeated measure ANOVA (variation factors: composite, treatment and evaluation period) and Tukey’s test at 5% significance level.

## RESULTS

The means and standard deviations of color change (∆1 and ∆2) are presented in Table 2. No statistically significant difference (p>0.05) was found among the composites before polishing (∆1). Color change in composites immersed in coffee was significantly higher (p<0.05) than that in composites immersed in the other solutions.

**Table 2:** Means (standard deviations) for ∆E values (ANOVA, Tukey's test; p<0.05)

**Treatments**		**Before Polishing (∆E1)**			**After Repolishing (∆E2)**	
	**Esthet-X**	**Surefill**	**Z-250**	**Esthet-X**	**Surefill**	**Z-250**
						
Distilled water	0.66(0.19)^a,A^	0.33(0.08)^a,A^	0.38(0.14)^a,A^	0.45(0.23)^a,A^	0.45(0.37)^a,A^	0.46(0.21)^a,A^
Coffee	3.67(0.64)^b,A^	3.57(0.98)^b,A^	4.85(1.33)^b,A^	1.53(0.39)^b,B^	2.35(1.09)^b,B^	2.29(0.76)^b,B^
Coca-Cola®	1.10(0.26)^a,A^	0.79(0.40)^a,A^	0.81(0.16)^a,A^	0.62(0.14)^a,A^	0.61(0.28)^a,A^	0.94(0.25)^a,A^

Different lowercase letters in columns and uppercase letters in rows indicate statistically significant difference at 5%. P matching value = 0.0087.

After repolishing, a significant decrease (p<0.05) in ∆ values was observed only for specimens immersed in coffee, while no significant change was observed.

## DISCUSSION

Because of the increasing patient’s demand for improved esthetics, there has been a claim for the development of restorative materials with excellent esthetic properties. However, to be considered as clinically acceptable, the materials must not only provide an initial shade match, but also maintain an esthetic appearance over the years in the restored tooth. Therefore, stain ability may be considered as a significant criterion in the selection of a material for use in an esthetically critical area.

The present study assessed the color stability of different types of composite resins when submitted to the action of staining solutions present in widely consumed beverages. The first null hypothesis was partially rejected because coffee presented a significant staining capacity regardless of the composite resin.

Color change can be assessed both visually^31^ and by specific instruments. The methodology used in the present study is in accordance with previous studies^7,10^ that used spectrophotometry and the CIe L*a*b* coordinates system. The CIe L*a*b* system was chosen to evaluate color variation (∆) because it is well suited for small color changes determination^12^ and have advantages such as repeatability, sensitivity and objectivity.

Several authors have reported that ∆ values ranging from 1 to 3 are perceptible to the naked eye^14^ and ∆ values greater than 3.3 are clinically unacceptable^18^. Considering these concepts, the composite resins tested in the present study showed unacceptable color changes when stored in coffee (Table 2). Khokhar, et al.^12^ (1991) also verified a strong staining of indirect resins after storage in coffee for 48 hours. Coffee was chosen as a staining solution in the present study because it has shown a high capacity of staining anterior composite resins and natural teeth^10^. According to Guler, et al.^8^ (2005), the average time for consumption of 1 cup of coffee is 15 min, and among coffee drinkers, the average consumption is 3.2 cups per day. Therefore, 15 days of storage simulated consumption of the drink over 1 year.

The staining capacity of the coffee on the composites can be justified due to the staining susceptibility of composite resins that might be attributed to their degree of water sorption and the hydrophilicity of the matrix resin^16^. Composite resins that can absorb water are also able to absorb other fluids with pigments, which results in discoloration.It is assumed that water acts as a vehicle for stain penetration into the resin matrix^20^. Water sorption occurs mainly as direct absorption in the resin matrix. The glass filler particles do not absorb water into the bulk of the material, but can absorb water onto the surface. Therefore, greater amount of resin matrix results in greater water sorption and weaker bonding between the resin matrix and filler particles in the composites. Further water sorption may decrease the durability of composite resins by expanding and plasticizing the organic matrix as well as by hydrolyzing the silane. The presence of microcracks into the resin matrix as a result of swelling and plasticizing effects along with interfacial gaps created between filler and resin matrix allow stain penetration and discoloration of the restoration^3^.

The affinity of the resin matrix for stains is modulated by its degree of conversion^6^ and by some physical properties, such as water sorption^3,16^. It seems that water sorption^16^ of composite resins depends on the composition of resin matrix used. It has been reported that water uptake in BisGMA-based composite resins increased from 3 to 6% as the proportion of TeGDMA increased from 0 to 1%^11^. UDMA seems to be more stain-resistant than Bis-GMA^12^. Under normal curing conditions, UDMA-based composite resin presented lower water sorption^18^ and higher color stability^12^ than having other dimethacrylates in their resin matrix.

The amount of unreacted monomer is directly dependent on the degree of conversion. Higher monomer conversion indicates low amount of unreacted monomer, lower solubility^23^ and higher color stability. Degree of conversion of composite resins light-cured under identical conditions ranges according to concentration of some monomers as some monomers present lower degree of conversion than others in the following order: Bis-GMA < BiseMA < UDMA < TeGDMA^22^. However, in the present study, these differences were not evident, as no significant difference in color change was noted among the tested composites, and all composites presented color alteration only when immersed in coffee.

Cola drink does not appear to be strongly implicated in color change of composites, despite the presence of phosphoric acid. Acids behave differently in promoting dissolution and hence in eroding the materials. In addition, the presence of phosphate ions in Coca-Cola^®^ may suppress the dissolution since these ions have been shown to reduce the dissolution rate of calcium phosphate from the tooth^1^.

Recently, manufacturers are producing composites with smaller filler particles in an attempt to produce materials with similar surface smoothness as that of dental enamel. An increase in filler content produces smoother surfaces because of the lower particle size and better distribution within the resin matrix^17^. When composite resins with different filler particle sizes were compared, higher ∆ values were obtained for hybrid composite resins compared to other types of composite resins. It has been reported that increased particle size resulted in less color change due to a decrease in the proportion of organic filler matrix^15^.

The different composite resins tested in this study did not show statistically significant change in color after immersion in distilled water and cola, but significant color change was observed after immersion in coffee. It has been reported that composite resins with a lower amount of inorganic fillers presented more color change because the greater resin matrix volume allows greater water sorption^7,11^. Vichi, et al.^29^ (2004) also observed a greater color stability for Z-100 (66% of inorganic particles in weight) when compared to Tetric-Ceram (60%) and Spectrum TPH (57%).

It is important that the composite resin presents a uniform distribution of filler particles in the polymer matrix and minimize the formation of filler-rich and filler-depleted areas within the composites. This is especially important with respect to the performance of composites in aqueous environments, since voids or nonbonding spaces at the filler/matrix interface may increase the water sorption of composites^24^.

In the present study, the high-density hybrid composite resin (SureFil), with greater inorganic volume (65%), showed the lowest ∆ values for all tested solutions when compared to the other composite resins, but this difference was not statistically significant (Table 2).

The 15-day test period was selected because most part of staining occurs after 1 week of immersion in coffee, and the following week serves as stabilization of the discoloration^10^. After 2 weeks of immersion, staining of the specimens and stabilization of the discoloration were observed in the present study. Different storage periods of specimens in staining solutions, such as 1 week^7^ , 48 h^12^ and 4 months^28^ have been reported.

A previous study^9^ on the finishing of resin-based restorations showed that highly smooth surfaces could be obtained when restorations were allowed to polymerize in contact with Mylar strips. However, it is usually necessary to remove excess material after placement of a restoration. According to Fontes, et al.^7^ (2009), the pigmented layer of the composite (approximately 40 µm) or the absorbed stains could theoretically be removed by polishing. Liporoni, et al.^13^ (2003) suggested that the discoloration of resin veneers can be partially removed by in-office bleaching and repolishing procedures. In this study, repolishing of the specimens’ surface stained by coffee was able to significantly reduce the ∆ values for the three tested composite resins, so the second tested null hypothesis was partially rejected.

Esthet-X showed a greater decrease in the ∆ mean values after repolishing, a reduction of 59% of discoloration, followed by Filtek-Z250 with a reduction of 53%, and lastly, SureFil with a 35% reduction of discoloration. These results could be attributed to the polishability and wear resistance of composites. Small filler particles allow wear^26,27^ ; thus, repolishing would be more effective in composite resins more susceptible to wear. Esthet-X, which has micro-inorganic fillers ranging from 0.04 to 1 μm^4^, presented lower staining values, because a greater number of particles were probably present on the surface. Consequently, a larger contact area may have been established between the fillers and the polishing system, resulting in improved reduction of discoloration.

## CONCLUSIONS

Under the tested experimental conditions, the following conclusions may be drawn: 1. The immersion of specimens in coffee caused a significant color change in all types of tested composite resins; 2. There was no significant difference of staining among the different types of tested composite resins; 3. Repolishing after staining reduced significantly the ∆ values of specimens immersed in coffee to clinically acceptable levels (∆<3.3).
